# Supplementary material for: Features of “false positive” unruptured intracranial aneurysms on screening magnetic resonance angiography
Source: PLoS One. 2020 Sep 3;15(9):e0238597. doi: 10.1371/journal.pone.0238597 (PMC7470380; doi:10.1371/journal.pone.0238597)
Supplement: Attachment — Submitted filename: Authors revesion letter.docx [file pone.0238597.s002.docx]

*Dear Academic editor of PLoS One*

*We would like to express our sincere appreciation to the reviewers and the editor for all the insightful comments that helped us in improving our manuscript. Please find following our point-by-point responses to the reviewers’ concerns. As mentioned in cover letter, the first decision was rejection, but the reviewers’ comments were somewhat modifiable. We revised the manuscript and hope that the corrections are satisfactory, and the revised manuscript could be undergoing re-consideration of yours.*

*Best regards,*

*from corresponding author of manuscript*

*This is the decision letter from you. The answer from ours is on Italic letters.*

**PONE-D-20-05254
Factors Associated with Digital Subtraction Angiography-Negative Unruptured Intracranial Aneurysms
PLOS ONE

Dear Dr. Kim,

Thank you for submitting your manuscript to PLOS ONE. After careful consideration, we have decided that your manuscript does not meet our criteria for publication and must therefore be rejected.

Specifically:

Several issues highlighted by the reviewers limits the possibility of considering the article for publication, especially regarding rationale, methodological and analysis concerns.

I am sorry that we cannot be more positive on this occasion, but hope that you appreciate the reasons for this decision.

Yours sincerely,

Miguel A. Barboza, MD, MSc
Academic Editor
PLOS ONE**

*Thanks for your thoughtful comment. We really appreciate you as well as respect your decision. However, the comments from three reviewers could be modifiable with efforts and it might be better strengthened in terms of rationale, methodological and analysis concerns.*

*As journal proposed accelerating the pace of scientific advancement and demonstrating its value on basis of scientific validity, rigorous methodology, and high ethical standards, with the aim of making all well-conducted research freely available, we sincerely thought that our submission is fit for PLoS One.*

**Review Comments to the Author**

**Reviewer #1: Thank you for the opportunity to review this interesting manuscript.
The text contains syntax and grammar issues that need fixing but still contains all the required information for methodologically evaluating it.**

*First of all, we really appreciated your insightful comments and concerns about the manuscript. We tried to fix some grammar errors and revised methodological misses. Please find the revised manuscript and check it again.*

**Besides the grammar aspects, there are other observations that I would like to suggest that the authors address before advancing in the editorial process:
The use of the following terms as synonyms across the manuscript: Digital Subtraction Angiography-Negative Unruptured Intracranial Aneurysms, "DSA-negative" aneurysms, unruptured intracranial aneurysms, DSA-negative aneurysms, false aneurysm, "DSA-negative (false)" aneurysms and DSA-negative (false) aneurysms. The clarification of the object of study is pivotal in evaluating the results. It is worth noting that one cannot talk about an aneurysm in the absence of an aneurysm, in this study once DSA becomes available and acts as the gold standard, there is no doubt regarding whether there is an aneurysm or not. Therefore, the use of the term false aneurysm, true aneurysm or any other name is confusing.**

*We totally agreed with your thoughtful comments. Our article tried to figure out the characteristics of the “false positive aneurysms on screening test of MRA or CTA”, that are finally confirmed as negative on DSA. In other words, we tried to investigate suspicious lesion of “aneurysm-like structure” on screening, but it was false positive that there was nothing aneurysm-like structure on DSA.*

*So, we clarified the main key word of the article in introduction and revised in whole of the manuscript. Also, we change the title as: “Features of False Positive Unruptured Intracranial Aneurysms on Screening Tests of Magnetic Resonance or Computed Tomographic Angiography That Are Finally Confirmed as Negative on Digital Subtraction Angiography”. “FPAS (false positive aneurysm on screening)” is newly defined and the study focused on securing the factors associated with FPAS.*

*Following sentences are in introductions: As screening angiography has been widely used, clinicians frequently encounter “suspicious aneurysm-like structures”, especially when they are too small or have a wide neck morphology. Following DSA is necessary to clarify these structures whether they are true aneurysm or not. These suspicious structures could be diagnosed with either “True positive aneurysm” or “False Positive Aneurysm on Screenings (FPAS)” depending on the results of DSA.*

**Another issue is that a false aneurysm (pseudoaneurysm) consists of an arterial wall defect that does not affect all three elements of the arterial wall. And the most common etiology leading to its formation is trauma, related to either catheter-based interventions or injury. Hence the need for clear definitions.**

*We agreed with your concerns that we used confused terms without considering the readers’ understanding. We tried to figure out the clinical features of false “positive” aneurysm, not false aneurysm. False aneurysm accompanies the pathological defect of arterial wall and the study did not intend this theme. So, we corrected the wrong words as your guidance.*

**The authors should provide the cause for investigating aneurysm in their patients. Were they asymptomatic, and the images categorized as aneurysms in non-invasive neuroimaging were therefore incidental? Or were they patients with definite subarachnoid hemorrhage (SAH) or with high suspicion of SAH?**

*We generally agreed with your observations. In methodological section, we clarified that unruptured intracranial aneurysm under 5mm size on screening is enrolled. In patients with subarachnoid hemorrhage or high suspicion of SAH, DSA should be mandatory. This situation is much different from general process of incidental aneurysm work-up. We just want to select true positive aneurysms on screening settings for reducing the un-necessary DSAs.*

*Actually, we also thought that the readers or reviewer might concern about the wide indication of the DSA (with high false-positive rates of small suspicious aneurysm-like structures). Maybe you are already aware of our affiliations, the study was performed in a single military hospital. Major patients in our hospital were young male soldiers or officials. Further, every military member should check their physical grade every other year, and the presence of intracranial aneurysm is important in their grading system. Especially, when patient have a true aneurysm in his or her cranium, they could discharge the military service. Consequently, a lot of patients with “suspicious” aneurysm-like structure are willing to undergo DSA in spite of its invasiveness. In discussion section, we already wrote about our situation as follows:*

*One major discrepancy between our results and that of previous studies was in terms of the false-positive rate. As mentioned, the sensitivity and specificity of screening tools for detecting UIA has been reported to be approximately 90%.^21-23^ However, in our cohort, the precision rate of detection of UIA was only 57%, and the false-positive rate was 43%. These differences occurred due to the inclusion of small aneurysms. In our hospital, the majority of patients with small aneurysms underwent confirmative angiography because they were in the military. In the manual of the physical grading system of the Republic of Korea Army, military personnel who have suspicious intracranial aneurysms are required to undergo catheterized angiography for a definitive diagnosis. While some may criticize such liberal guidelines regarding cerebral angiography in military hospitals, it is necessary for allocating personnel to the appropriate department. Furthermore, our significant experience in performing catheterized angiography for small aneurysms is informative and have enabled the current study design. Further investigations enrolling smaller aneurysms are expected to reveal sensitivity and specificity of CTA/MRA similar to that in our study.*

**I strongly advise the use of standard terminology for intracranial arteries, especially for the internal carotid artery since "the communicating internal carotid artery (ICA)" is not a known artery nor segment of the ICA. I believe that what the authors are trying to describe is the communicating (terminal) segment of the ICA, also known as C7 in the Bouthillier classification.**

*Thanks for your concerns. We do follow the Bouthillier classifications. We revised the whole manuscript with careful observations. Also, the term of “Paraclinoid” is not a standard, we fixed this word to C5-6 of the Bouthillier classification.*

**Regarding the main analysis, it seems redundant to include in the same regression a surrogate of aneurysm measure (aspect ratio) along with a true measure that is already included in the surrogate (height). The authors should also be aware of the low count of cases for some of the variables such as cavernous ICA and distal ACA locations. These low counts have a significant effect on the behavior of the statistical model.**

*Thanks for your kind reviews. In terms of low count of cases, we agreed with your comments and conducted analysis again. We simply the location of aneurysm as C5-6, C7, and others according to the incidences. Also, minor parameter of “aspect ratio >1” and “dome to neck ratio >1” was erased. The result was similar that location on C7 ICA showed significance. Instead, height and wide neck morphology (dome to neck ratio <0.99) were significant. In terms of Aspect or Dome to Neck ratio, which were calculated from original value of height, neck, and width, it has different meaning after secondary calculations. We agreed that there might be some confounding effects. However, dome to neck ratio and height were finally identified as significant in multivariate study, which statistically confirms after correcting the confounding effects. The interpretation of the results would be more reliable regardless of confounding matters.*

**I suggest the use of cut point values instead of the term Youden index. Otherwise, the authors need to provide the actual index values.
Finally, the provided text and data do not support the authors' conclusion; this study does not address the follow-up of patients.**

*As your concerns, we modified the term of “Youden index” to “cut point values”. Also, we agreed with your comments that the conclusion is less logistic. We changed as follows: Suspicious aneurysm-like structures, which are located on the C7 ICA, have a wide neck morphology (dome to neck ratio < 0.99), and are small (<2.4 mm in height), can be highly suspected as negative on DSA.*

**Reviewer #2: A couple of sentences and affirmations merit an specific response:
1. “a true aneurysm can be highly suspected in the initial screening, clinicians occasionally encounter. DSA-negative (false)” aneurysms, especially when the aneurysm is too small or has a wide neck and small height”:
RE: DSA is the gold standard: what is positive in DSA is truly positive (>99%) . What it is negative in DSA is truly negative (99% of the cases). If an UIA is falsely detected by a particular noninvasive screening neuroimaging (MRA-CTA) modality and the UIA is subsequently ruled out by DSA, it is a false positive problem of the first modality and a true negative (not false negative) finding of the second one.**

*First of all, we really appreciate your concerns for strengthening the manuscript. We totally agreed with your thoughtful comments. Our article tried to figure out false positive aneurysms on screening test of MRA or CTA, that are finally confirmed as negative on DSA. In other words, we tried to investigate suspicious lesion of “aneurysm-like structure” on screening, but it was false positive that there was nothing aneurysm-like structure on DSA. So, we clarified the main key word of the article in introduction and revised in whole of the manuscript. Also, we change the title as: “Features of False Positive Unruptured Intracranial Aneurysms on Screening Tests of Magnetic Resonance or Computed Tomographic Angiography, That Are Finally Confirmed as Negative on Digital Subtraction Angiography”. “FPAS (false positive aneurysm on screening)” is newly defined and the study focused on securing the factors associated with FPAS. For example, following sentences are in introductions: As screening angiography has been widely used, clinicians frequently encounter “suspicious aneurysm-like structures”, especially when they are too small or have a wide neck morphology. Following DSA is necessary to clarify these structures whether they are true aneurysm or not. These suspicious structures could be diagnosed with “True positive aneurysm” or “False Positive Aneurysm on Screenings (FPAS)” depending on the results of DSA.*

**2. “clinicians can now manage small aneurysms that were not recommended for treatment in the past”
RE: Aneurysm should be treated according evidence based indications not according how easy they are to be treated.**

*Thanks for your insightful comments. We agreed that aneurysm should be treated according to the evidences. In prospective RCT of ISUIA study, aneurysm under 7mm in size could be observed with regular follow-ups. However, a lot of rupture cases were reported in aneurysm smaller than 7mm, and safety of endovascular and surgery has already been anticipated. As your comments, we changed the introduction as follows: However, small aneurysms, which were not recommended to be treated in the past, are widely accepted as treatment indications according to recent evidences.^5,6^*

**General population scrutiny for UIA is not indicated. Unlikely this population presented din this study, UIA are more prevalent in women. Screening for UIA would be indicated when the subject has 2 or more than 2 first or second degree relative with intracranial aneurysm. This is particularly relevant for women. Familial intracranial aneurysm syndrome is tends to pair between mothers and daughter and siblings (sisters). In this skewed sample of male soldiers ¾ of the aneurysm were located at the communicating segment of the IC ICA. This a particular troublesome spot for unruptured small intracranial aneurysm because the presence of the takeoff of the posterior communicating artery. The Pcomm is responsible for the posterior circulation blood flow in the fetal stage. Infundibular dilatation are relatively common in the general population and frequently misinterpreted in non-invasive neurovascular studies as UIA. Infundibulum tend to do not grow over time and the risk of rupturing is negligible. Occasionally, they can give rise to infundibular aneurysmatic dilatations. The risk of rupturing of this aneurysmatic dilatation is also suspected to be extremely low.
On the other hand aneurysm less than 3 mm in such us low risk locations like anterior circulation do not need invasive confirmatory studies. A 6-18 months fup CTA or MRA would be more than enough.**

*We appreciated your thoughtful comments. We agreed that general population scrutiny for UIA is not indicated. We do not intend the investigation about better tools for screenings or selection for patient indicated for screenings. Instead, we tried to identify the clinical or radiologic features of small aneurysm-like structure that are positive on screening angiography and finally false negative on DSA. We occasionally met these aneurysm-like structures in out-patient clinics and this might be the challenging situation whether we should perform DSA. By analyzing our data, we just tried to provide a “clue” for reducing the un-necessary DSA performances.*

*Further, in terms of enrolled population, we do agree with your concerns. It is far different from general populations that the majority of ours is young, male, soldiers. However, the location or geometry of an angiographic finding is the most important parameters in analyzing and population difference may less affect the results. As your concerns, we wrote the limitation of difficult interpretation of the enrolled populations: Third, the majority of the enrolled populations are young, male, soldiers, that have differences from general populations. Although the geometry and location of the aneurysm-like structures are the key parameter of the current study, the interpretation of the result needs cautions.*

*Finally, in terms of PCoA infundibulum, we agreed with your comment. Maybe, the result (location at C7 ICA) is a matter of course. Instead, we unpack the reason of the result why these structures look like aneurysm on MRA or CTA in discussion section. For example, on MRA, slow flow or flow from a vessel parallel to the scan plane may become desaturated as that with stationary tissue, resulting in signal loss from the vessel. Therefore, small PCoA flow disappeared in TOF images. These analyses might help physicians when they decide whether perform DSA or not.*

**Reviewer #3: In general it is an interesting subject, however I have some observations.
1. I think it is necessary to define "DSA negative" "DSA false" and “false anevrysms”, as it is confusing at the time of reading.**

*Initially, we really appreciated your reviews which might be corrected for better understanding the manuscript. We agreed with your concerns, what other reviewers also raised. Our article tried to figure out false positive aneurysms on screening test of MRA or CTA, that are finally confirmed as negative on DSA. In other words, we tried to investigate suspicious lesion of “aneurysm-like structure” on screening, but it was false positive that there was nothing aneurysm-like structure on DSA. So, we clarified the main key word of the article in introduction and revised in whole of the manuscript. Also, we change the title as: “Features of False Positive Unruptured Intracranial Aneurysms on Screening Tests of Magnetic Resonance or Computed Tomographic Angiography, That Are Finally Confirmed as Negative on Digital Subtraction Angiography”. “FPAS (false positive aneurysm on screening)” is newly defined and the study focused on securing the factors associated with FPAS. For example, following sentences are in introductions: As screening angiography has been widely used, clinicians frequently encounter “suspicious aneurysm-like structures”, especially when they are too small or have a wide neck morphology. Following DSA is necessary to clarify these structures whether they are true aneurysm or not. These suspicious structures could be diagnosed with “True positive aneurysm” or “False Positive Aneurysm on Screenings (FPAS)” depending on the results of DSA.*

**2. In Table 2, Angio-negative and Angio-positive is equal to DSA negative and DSA positive? if the answer is yes, it might be better to unify the terms.**

*We agreed with your comments. In flow chart, we divided the enrolled “suspicious aneurysms-like structures” on screening angiography into “true positive” and “false positive aneurysms” groups according to the result of DSA. So, in Table 2, we modified the group names as (1) False positive, and (2) True positive.*

**3. In the table 2, please verifier the number of MRA and CTA reported.**

*Thanks for your concern. We found the errors and fixed them.*

**4. In comparative studies of CTA and DSA, aneurysms of 3 mm and smaller are commonly missed with CTA, but you have taken as criteria for inclusion aneurysms smaller than 5 mm, why did you choose that value?**

*We generally agreed with your comments. It is generally agreed that aneurysms under 3mm (or smaller) would be negative and have low risk for rupture. However, whether we should perform DSA or not is another problem. There is no definitive guideline about the selective indication of performing DSA. It would be the physicians’ choice and the finding of current study might help for guidance.*

*Further, we selected the enrolled aneurysm under 5mm. While there were some other features of rupture prone characteristic, such as accompanying daughter sac, high aspect ratio, or enhancement on high resolution vessel wall MRI, it is generally accepted that aneurysms under 5mm in size have low risk for rupture and therefore it can be followed up with non-invasive angiography. We tried to strengthen this concept with evidences by identifying the clinical or radiologic features of small aneurysm-like structure that are positive on screening angiography and finally false negative on DSA. So, we enrolled the small aneurysms under 5mm.*

**5. While the factors you found can help make a decision in following up with patients, usually in small unruptured aneurysms (UIAs) the risk of rupture is low, so they do not require treatment, therefore an invasive confirmatory study or treatment is questionable.**

*We agreed with your comments. Generally, these small suspicious structures can be observed with non-invasive follow-up. However, the number of DSA cases is increasing regardless of its size, and the guideline of selective DSA would be mandatory in near future. Further, maybe you are already aware of our affiliations, the study was performed in a single military hospital. Major patients in our hospital were young male soldiers or officials, and every military member should check their physical grade every other year. The presence of intracranial aneurysm is important in their physical grading system. Also, in insurance problems, some patients want to undergo the confirmative DSA regardless of its size. So, in this special situation, our finding may help physicians’’ decision whether DSA should be performed or not.*
